# Feasibility of a complex psychosocial intervention for families with parental cancer: acceptability, suitability, implementability, and perceived support

**DOI:** 10.1007/s00432-024-05946-5

**Published:** 2024-10-17

**Authors:** Nicole Ernstmann, Hannah Nakata, Lina Heier, Christian Heuser, Marc Dohmen, Rebecca Bremen, Franziska Geiser, Steffen Holsteg, Andre Karger, Anja Viehmann, Manuela Brüne, Andrea Icks, Burkhard Haastert, Tim H. Brümmendorf, Andrea Petermann-Meyer

**Affiliations:** 1 Chair of Health Services Research, Institute of Medical Sociology, Health Services Research and Rehabilitation Science, Faculty of Medicine and University Hospital Cologne, University of Cologne, Eupener Str. 129, 50933 Cologne, Germany; 2 University Hospital Bonn, Center for Health Communication and Health Services Research, Department of Psychosomatic Medicine and Psychotherapy, Bonn, Germany; 3 Center for Integrated Oncology Aachen, Cologne and Düsseldorf (CIO ABCD), Bonn, Germany; 4 Department of Clinical Pharmacy and Toxicology, Maastricht University Medical Center, Maastricht, Netherlands; 5 CARIM School for Cardiovascular Disease, Maastricht University, Maastricht, Netherlands; 6 Department of Hematology, Oncology, Hemostaseology and Stem Cell Transplantation, Faculty of Medicine, RWTH Aachen University, Aachen, Germany; 7 University Hospital Bonn, Department of Psychosomatic Medicine and Psychotherapy, Bonn, Germany; 8 Clinical Institute of Psychosomatic Medicine and Psychotherapy, Medical Faculty, Heinrich-Heine University Düsseldorf, University Hospital Düsseldorf, Düsseldorf, Germany; 9 Institute for Health Services Research and Health Economics, Centre for Health and Society, Medical Faculty, Heinrich-Heine-University Düsseldorf, University Hospital Düsseldorf, Düsseldorf, Germany; 10 mediStatistica, Wuppertal, Germany

**Keywords:** Complex intervention, Feasibility, Implementation, Parental cancer, Children

## Abstract

**Purpose:**

This study aimed to assess the feasibility of a comprehensive psychosocial intervention for families coping with parental cancer.

**Methods:**

A quasi-experimental trial with intervention and control group, employing a mixed-methods approach, was conducted. A total of 472 families affected by parental cancer participated. The feasibility of the intervention was evaluated based on study monitoring measures (on-site visits, team supervision meeting observations, case conference observations, best practice workshops, coordinating information exchange between intervention sites, and reviewing intervention documentation), process evaluation (semi-structured interviews, focus group discussion) and survey data. Data analysis involved thematic coding and descriptive statistics.

**Results:**

The intervention was well-received by the participating families, with a high degree of acceptance observed. The feasibility of the intervention was found to be associated with specific dynamics within each family system and the motivation of the family members. The success of the intervention was described as dependent on the family-centered arrangement of the encounters, including factors such as frequency, duration, and mode, which greatly influenced its overall acceptability.

**Conclusion:**

The family-scout intervention demonstrates its feasibility as an effective intervention to reduce the burden experienced by families coping with parental cancer. Psychosocial oncology services should continue to develop and implement family-centered interventions to offer support to families during their cancer journey.

**Trial registration:**

ClinicalTrials.gov, NCT04186923. Retrospectively registered on 4 December 2019.

Eupener Str. 129, 50933 Cologne, Germany, nicole.ernstmann1@uk.koeln.de, https://orcid.org/0000-0001-7685-6110.

## Introduction

All family members are affected by a parental cancer diagnosis. Patients often suffer from anxiety, depression and psychological or social distress (Herschbach et al. 2020; Mehnert et al. 2018) even 5–10 years after diagnosis (Götze et al. 2020; Breidenbach et al. 2022). Younger patients with minor children have more often a need for psychosocial support than patients without minor children, even several years after diagnosis (Hammersen et al. 2023). Partners of cancer patients suffer from anxiety, depression and distress and show increased risks for morbidity (Götze et al. 2017; Rosenberger et al. 2012) especially in palliative care situations (Oechsle et al. 2013) and after the possible death of the parent (Falk et al. 2021). Children of cancer patients have a higher risk of anxiety, depression, and psychosomatic complaints (Ernst et al. 2015); however psychosocial interventions often do not meet children’s needs adequately (Ohan et al. 2020). In case of a patient´s death teenagers might hide their grief, as they do not want to burden the grieving parent (Sveen et al. 2016). Experiencing parental cancer during childhood predicts higher anxiety levels during young adulthood (Metcalf et al. 2017).

Parents have difficulties in understanding their children’s reactions (Hauskov Graungaard et al. 2023) and tend to underestimate the impact of their being affected by cancer on their respective children (Morris et al. 2016). When parents observe signs of distress in their children, they often avoid communication about emotional reactions though parent-child communication and open cancer communication are key elements in supporting children’s coping (Hauskov Graungaard et al. 2023). Research indicates that couples coping with cancer tend to react as an emotional system rather than as individuals (Götze et al. 2017). Hence, the parental cancer is a burden for the family as a unit. However, often parents lack family-centered psychosocial support (Inhestern et al. 2021; Hauskov Graungaard et al. 2023; Johannsen et al. 2023). A high frequency of unmet family-centered support needs in diverse aspects have been shown (Hammersen et al. 2021).

Family adjustment and resilience when experiencing parental cancer is associated with the adaptability to changes, use of social resources, family cohesion, mutual support, flexibility, open communication, and psychological well-being (Perak et al. 2024; Heuser et al. 2024). To strengthen family resilience and to reduce the psychological burden of all family members the complex intervention family-scout has been developed (Petermann-Meyer and Hugot 2022). The intervention components (Hoffmann et al. 2014) are active outreach support, permanent contact person, psychosocial needs assessment, communicative as well as emotional and organizational support, family-centered and child-centered therapeutic interventions, networking within the healthcare system, educational system, and the youth welfare system. The intervention is provided by social workers. It is delivered for a period of up to nine months via home visits, telephone support, or video calls. Receivers are children, spouses, cancer patients, in an individual, couple, family setting or depending on the targeted constellation. Type and number of interventions, i.e. frequency, duration, and thematic focus of the encounters depends on the family members needs and preferences. In case of lower psychosocial needs, the family members will be offered four encounters, e.g. to establish household help or support open family communication. In case of higher psychosocial needs, the family members will be offered support until a substantial decrease of burden will be reached or until family members decline ongoing support. In case of a need for specialized psychooncological or familial support beyond the family-scout’s ability, the participating families will be referred to cooperating psychotherapists trained in psychooncological and/or family interventions (Romer et al. 2014). During the social distancing restrictions caused by the COVID-19 pandemic, the family-scout intervention has been amended by video calls.

First evidence for the effectiveness of the family-scout intervention has been found in the main study (Petermann-Meyer et al. 2024). However, evidence to the acceptability, suitability, or implementability of the family-scout intervention is still missing. There are some insights from implementation research on similar interventions. A German study on the implementation barriers of a psycho-oncological family intervention has shown that it is necessary to provide physicians with evidence of the value of the intervention since families are more likely to utilize the service when it is referred to by their physicians (Romer et al. 2007). Patients’ fears of psychiatric stigmatization should be carefully addressed (Romer et al. 2007). A systematic review on barriers of implementation and use of psychosocial interventions for families affected by parental cancer found practical difficulties, perceived need for support or fear of stigma as family-related factors (Inhestern et al. 2016). The review showed that intervention characteristics such as a flexible structure are important facilitators of implementation (Inhestern et al. 2016). An implementation study on a psycho-oncological intervention for couples coping with cancer showed that the successful implementation strategies included identifying values-benefits-outcomes for key stakeholders, recruiting an interdisciplinary team, utilizing a stepwise implementation model, integrating the intervention into existing institutional processes, and collecting data and credibility (Bitz et al. 2020). In accordance, a qualitative Australian study on implementation barriers of oncology social work showed that a lack of understanding of the social work role by clients and colleagues is a common barrier indicating a need for improved integration of psychosocial care into overall patient engagement with the cancer center (Pockett et al. 2016).

The aim of this feasibility study is to analyze the acceptability, suitability, and implementability of the family-scout intervention in three university hospitals being sites of a comprehensive cancer center.

## Methods

Following the Medical Research Council guidance, the family-scout intervention has been developed according to empirical evidence, such as the specific support needs of affected families, and a theoretical framework (Romer 2007; Romer et al. 2014) and tested in a monocentric pilot study (Krüger et al. 2023). In a next step, the multicentric effectiveness-implementation hybrid study (Curran et al. 2012) has been conducted to evaluate the effectiveness of the family-scout intervention and to assess its implementability during the clinical evaluation stage (Klaic et al. 2022). The study used a mixed method quasi-experimental design with intervention (IG) and control group (CG). A standardized family survey at three measurement points (T0: study enrollment; T1: 3 months follow-up; T2: 9 months follow-up), claims data analysis, and semi-structured interviews were conducted for summative and process evaluation. The study design and methods have been described elsewhere (Dohmen et al. 2021). The study has been approved by the ethics committees of the participating centers.

The feasibility analysis presented here is part of the process evaluation within the effectiveness-implementation hybrid study. The RE-AIM framework (Damschroder et al. 2009; Glasgow et al. 1999) was used to guide the analysis. It includes the assessment of reach, effectiveness, adoption, implementation, and maintenance. We focused on adoptability and implementability as anticipated implementation outcomes (Damschroder et al. 2022). We added the assessment of individual aspects to the assessment of rather organizational aspects as originally intended by Glasgow et al. (Glasgow et al. 1999). To analyze aspects of adoptability and implementability of the intervention, we adapted the criteria of Orsmond and Cohn (Orsmond and Cohn 2015) as they not only define the main objectives of a feasibility study but also provide useful guiding questions that can lead the analysis. The analysis followed the guiding questions of “objective 3” (evaluation of acceptability and suitability of intervention), “objective 4” (evaluation of resources and ability to manage and implement the intervention), and “objective 5” (preliminary evaluation of participant responses to intervention). We used the following four data sources for our comprehensive approach to feasibility analysis.

### Acceptability, suitability, and implementability of intervention

1) Data gathered in the intervention monitoring process served as data source for the assessment of the acceptability, suitability, and implementability of the intervention. Monitoring steps included on-site visits, remote monitoring activities (including phone calls, video visits, e-mails), intervention documentation checks, semi-structured feasibility interviews (see below), chairing best practice workshops, observations of patient recruitment, patient screening, patient enrollment, team supervision meetings, and case conferences, as well as coordinating exchange between the intervention sites (see Table 1). The data basis consisted of a comprehensive monitoring table in which the monitor noted problems and challenges that were raised during the monitoring steps. The topics were discussed in the regular meetings of the evaluation team and the proposed measures were documented in the table. It was also recorded for each measure how it was reported back to the study management and the family-scouts and which proposed solutions were implemented.

**Table 1:** Intervention monitoring procedures

Group	Monitoring focus	Monitoring activities
familiy-scouts	− acceptability, suitability − treatment fidelity − intervention implementation resources	− on-site visits − remote monitoring activities − observations of team supervision meetings − observations of case conferences − chairing best practice workshops − coordinating exchange between the intervention sites − intervention documentation checks − semi-structured feasibility interviews − focus group discussion
project managers	− fidelity to research protocol − intervention implementation resources	− on-site visits − remote monitoring activities − observations of patient recruitment − observations of patient screening − observations of patient enrollment − focus group discussion
principal investigator	− development of intervention manual − training of family-scouts − development of criteria for assessing treatment fidelity − intervention implementation resources	− on-site visits − remote monitoring activities − semi-structured feasibility interview − focus group discussion
research team	− adverse events reported in the questionnaire survey	− on-site visits − remote monitoring activities

2) Six semi-structured feasibility interviews with family-scouts were conducted and audiotaped. All family-scouts were female and had more than five years of professional experience. The interview questions were based on the guiding questions by Orsmond and Cohn (Orsmond and Cohn 2015). The interviewer took field notes during and after the interview. The interview material was subject to single thematic coding by two researchers. The coding results were discussed and revised in a focus group with the interviewees, the project managers and the supervisor of one intervention site. The final results were fixed during the focus group discussion in a protocol.

### Preliminary evaluation of participant responses to intervention

3) Data collected from the standardized family survey at T1 (3 months follow-up) were utilized to assess the perceived support provided by family-scouts from the families’ perspective. The assessment of perceived support employed a three-item scale, which was adapted for family-scouts from the support by physicians scale. Respondents used a four-point Likert scale to indicate their level of agreement with statements related to supportive behavior, with response options ranging from 1 (strongly disagree) to 4 (strongly agree). The original items and their psychometric properties are detailed elsewhere (Ommen et al. 2009).

4) 23 semi-structured interviews were conducted with *n* = 16 partners in the intervention group. 7 partners were serially interviewed at two time points. The interview questions addressed the family´s daily life, the support needs, the family communication and the family-scout-experience. The interviews were audiotaped and fully transcribed. The mean duration of the interviews was 31 min. The interview material was subject to single thematic coding by three researchers (King 2004).

## Results

Overall, 915 families were screened and *n* = 472 families participated in the study (response rate 52%). The mean age of the participating cancer patients was 44,6 (IG) and 42,7 (CG); 317 of these were female. The mean age of the healthy partners was 46,0 (IG) and 43,0 (CG); 103 of these were female. The age range of children in the participating families was between 0 and 36 years, and the average number of children was 1.7. Detailed sample and baseline characteristics have been described elsewhere (Petermann-Meyer at al. 2024).

### Acceptability, suitability, and implementability of intervention

Results of the feasibility analysis from the monitoring and family scouts’ perspective are presented in Table 2 (acceptability and suitability) and Table 3 (implementability).

**Table 2:** Acceptability and suitability

Guiding Question	Data	Results
What factors affect the cooperation of families in the intervention/the adherence to the intervention?	− semi-structured feasibility interviews (family-scouts) − focus group discussion	− Tailoring the intervention in terms of content (communicative, emotional, and organizational support), intensity (frequency and duration of contacts), and form (home visits, phone calls, video calls) to the needs of the families is crucial for cooperation − The building of a trustful relationship between family-scout and family members and an initial mission clarification is a prerequisite for collaboration − The cooperation varies between families and between family members depending on o the families’ actual emotional burden and motivation of the family members o the family structure and actual needs o the disease status of the cancer patient and on the acceptance of the disease by the family members
Do the participants have enough time and capacity to benefit from the intervention?	− semi-structured feasibility interviews (family-scouts) − focus group discussion − observations of case conferences	− The intervention should be implemented as early as possible following the cancer diagnosis; families will benefit in later disease stages from an implemented family-scout but might be unable to start the intervention at this stage − In case the intervention is perceived as helpful and family-centered, the families take their time to complete the intervention − Phases of different time requirements can occur along the course of the disease − Time demands increase during phases of transition (e.g., rehab, reintegration, recurrence, death) − There may be long periods between contacts (3 or 4 months), yet families benefit from contacts − The key is flexible scheduling, adapting to family needs − Time required for initial interview is approx. 2 h, for home visits 1–2 h, for video calls approx. 1 h, for telephone calls 20–40 min − Home visits 1/month are feasible for most families
Does the intervention create a burden for the participants?	− semi-structured feasibility interviews (family-scouts) − focus group discussion − observations of case conferences	− The burden of the intervention depends on the intervention tailoring to the family’s needs − A neutral environment can be helpful for certain issues − It can be helpful to form protective spaces with different settings (children, parents, partners). − For very closed family systems with rigid external boundaries, home visits can be a burden. Opening up to a family-scout is challenging for closed family systems
To what extent is the intervention acceptable and appealing to participants?	− semi-structured feasibility interviews (family-scouts) − focus group discussion − observations of case conferences	− The degree of acceptance of the intervention in the families is high; most families are relieved to accept the offer − The intervention is appealing in terms of: o Flexibility (regarding time/working with different family members/topics) o Family-centeredness o One single coordinating and caring contact person o Client-centered communication on equal terms − For some families with dysfunctional structures, the openness of the intervention is especially attractive − Acceptance of family-scouts seems higher than acceptance of youth welfare or psychotherapy (no focus on deficits but resource orientation) − Acceptance depends on previous experiences with help systems − Acceptance depends on the external boundaries of the family, i.e. the degree to which the family is open for support from outside − Families with acute stress may find it harder to accept the intervention; families with less stress may need the intervention less − Families in palliative care situations may find it difficult to accept the intervention. At least two months before the death of the parent seem necessary for relationship building − Families in which there is less awareness of the needs and burden of individual family members tend not to be reached by the intervention − Acceptance problems arise when expectations are disappointed − Fear of some parents that their children might experience disadvantages due to an F-diagnosis (e.g., when taking out insurance, becoming a civil servant) − Support from youth welfare services is difficult to accept (especially for families who have experience with youth services) − No specific oncology attraction: families with other serious or chronic progressive illnesses of a parent might also benefit from the intervention
What is the level of safety of the procedures in the intervention?	− semi-structured feasibility interviews (family-scouts) − focus group discussion − observations of case conferences	− The level of safety for clients and providers is high − There were no adverse events observed that were attributable to the intervention − There was no early termination of the intervention, as the length of the intervention was not predetermined − However, some families terminated the intervention even though the family-scout still saw a need for support (e.g. in case of disappointed expectations or projection) − In case of multiple problems in the families (e.g. mental, social or employment related issues), the intervention was occasionally terminated by the family-scouts due to a lack of resources of the family-members to address the illness-related issues
Are there any unexpected adverse events?	− semi-structured feasibility interviews (family-scouts) − focus group discussion − on-site audits − audit calls	− There were no unexpected adverse events observed that were attributable to the intervention − Participants reported additional burden during the COVID-19 pandemic − One minor child reported (in the postal survey) anxiety due to threatening behavior of the parent associated with cancer side effects, not with the intervention − Some concerns regarding open communication in the family leading to exclusion of children or partners from encounters with the family-scouts

**Table 3:** Implementability

Guiding Question	Data	Results
Does the team have the administrative capacity, expertise, skills, space and time to conduct the intervention?	− semi-structured feasibility interviews (family-scouts) − focus group discussion − on-site audits − audit calls	− The project managers were responsible for the recruitment of the members of the professional network, information, maintaining contacts and for the coordination of the professional network − As part of the implementation process at other intervention sites, this step will have to be carried out by the family-scouts themselves − The time of approx. one hour needed for the inclusion of a family has to be considered − Ideally, teams of two family-scouts should be responsible for the care for each family − A full-time employed family-scout might be able to support a maximum of 50 families; part-time positions are recommended due to high emotional load − Emotional and organizational burden of the family-scouts is high when there is a lack of acceptance for the illness − Time resources are needed to keep the family-scouts’ expertise on socio-legal support up to date − Time resources for administrative case management have to be considered
To what extent does staff comply with the approved human participants’ protocol?	− semi-structured feasibility interviews (family-scouts) − focus group discussion − on-site audits − audit calls	− The family-scouts, project managers and the research team comply with the data protection protocol − Agreements to relieve the family-scouts from their obligations of confidentiality are obtained when necessary − The family-scouts report child welfare risks according to their legal obligation
How effectively are adverse events during implementation identified, documented, and reported?	− semi-structured feasibility interviews (family-scouts) − focus group discussion − on-site audits − audit calls	− Possible adverse events can be reported and discussed in case conferences − Possible adverse events can be documented in family records − In hospitals and cancer centers, errors or adverse events might be reported on a voluntary basis in anonymous Critical Incidence Reporting Systems (CIRS) − The family-scouts discussed their own distress in team supervision meetings and case conferences
Can the intervention be conducted within the designated budget?	− semi-structured feasibility interview (principal investigator) − focus group discussion	− During the F-SCOUT-trial o The intervention could be conducted within the designated budget o The experience gained in the pilot study helped plan the budget. Costs were determined by hourly rates o Intervention tailoring: There were no two categories with defined intervention doses in a period of max. 9 months. The family-centeredness led to a family-centered intervention intensity and length (incl. longer breaks for several months). On average, the fixed cost framework was sufficient − After the F-SCOUT trial (i.e. after the project funding by the Innovation fund of the German Federal Joint Committee) financing will continue on a transitional basis by o Selective contracts with the statutory health insurance funds according to § 140a social security code 5 with per-case reimbursement o Application of a modular solution that reflects the individual intervention intensity, duration and length are necessary
What technology and equipment is needed to conduct the intervention, including collection, management, and analysis of data?	− semi-structured feasibility interviews (family-scouts) − focus group discussion	− Office with personal IT equipment (Personal computer with MS Office, camera, microphone, speaker, printer, fax, possibly notebook) − Documentation software incl. master database of participants − telephone, cell phone − Consultation room, meeting room − Technical literature (social security code, diagnostic manuals) − Literature, brochures, guidebooks, material for children, adolescents, adults − Application forms − Company car/car sharing car or private car for official use, parking space − Public relations material (flyers, business cards, giveaways)
What is involved in training personal to use the equipment?	− semi-structured feasibility interviews (family-scouts) − focus group discussion − on-site audits − audit calls	− The family-scouts need MS office skills − Competencies in dealing with specific database solutions can be conveyed in short training courses or by consulting of the involved IT departments
Who are the members of the professional network needed to implement the intervention?	− semi-structured feasibility interviews (family-scouts, project managers and principal inverstigator) − focus group discussion − on-site audits − audit calls	− Providers: health insurance companies, youth welfare office − Health care sector: cancer centers, hospitals, family care/ welfare associations, specialists (e.g., oncologists, pediatricians), family physicians, psychooncologists, case managers, nursing services, social services, psychotherapists (individual, couples and families), palliative care, hospices, social pediatric centers − Youth welfare services − Cancer counseling centers − Lawyers (Inheritance/adoption/custody) − Housekeeping services − Kindergarten, school, vacation care − Municipal health departmens − Self-help groups − Debt counseling − Donors
Are there special qualifications needed in the professional network?	− semi-structured feasibility interviews (family-scouts, project managers and principal investigator) − focus group discussion	− Vocational training in social work plus o Knowledge/experience in oncology/nursing/psycho-oncology/hospice/health care system o Training in systemic counseling/family work or experience in family work o Reflection on the topic of death and dying − A mandatory training manual for F-SCOUT was developed with the following content o Psychosocial interventions o Social law, especially services according to the different social security code books (V, VIII, and XI) o Coping with cancer o Family communication and communication about death and dying o Developmental psychology: childrens’ coping with death and dying o Professional network o Basic oncological/medical knowledge and knowledge of rehabilitation
How long does the building and implementation of the professional network take?	− semi-structured feasibility interviews (family-scouts, project managers and principal investigator) − focus group discussion	− The duration depends on the a priori individual and professional network of the family-scouts, the project managers and supervisors − In case of no a priori networks the building of the network might take up to six months − The introduction of the intervention to the network partners can be done through meetings, visits, in-house trainings and written information − Primary care providers (physicians, psychooncologists, social workers, and nurses) should learn how to identify and reach out to families with minor children − Crucial for the implementation is the cooperation between the medical system and the child welfare system − Helpful for the implementation and maintenance of the network is a regular round table meeting with all stakeholders to connect the actors
How is financing of the intervention in routine care ensured?	− semi-structured feasibility interview (principal investigator) − focus group discussion	− Based on the evaluation of the primary and secondary outcomes, the Federal Joint Committee will decide about the reimbursement of the family-scout intervention by the statutory health insurance in Germany − The framework for the reimbursement can be a contract for integrated care according to § 140a, § 119, § 63 and § 64 of the social security code book 5 − As an interim solution until the evaluation of the primary and secondary outcomes have been performed, a contract for integrated care according to § 140a of the social security code book 5 has been signed by the participating university hospitals and some of the participating statutory health insurance companies − As interim solution, a package price is paid in three quarterly installments by the participating health insurance funds for the intervention
What is the most suitable organizational affiliation for the intervention team?	− semi-structured feasibility interviews (family-scouts, project managers and principal investigator) − focus group discussion	− Suitable/not suitable affiliation o The affiliation to a welfare organization cooperating with a comprehensive cancer center might be suitable o The affiliation to a comprehensive cancer center with a psycho-oncology department might be suitable o The affiliation to a cancer counseling center might be suitable o The affiliation to psycho-oncology/psychotherapy unit may deter some families o An organizational affiliation to the nursing service/case management/social service may not be suitable due to the limited therapeutic focus − Structures/functions needed within the organization o A psycho-oncology advisory function for family-scouts o A medical/oncological advisory function for family-scouts o A socio-legal advisory function for family-scouts o The option to use external case or team supervision o A supportive team climate

### Preliminary evaluation of participant responses to intervention

At T1, a total of *N* = 142 healthy partners in the intervention group completed the questionnaire, resulting in an 84% response rate. Of these respondents, 30% were female partners, and the average age was 46 years. The healthy partners provided an assessment of the perceived support: On a scale of 1–4 (4 = maximum perceived support), item means between 3,11 and 3,57 were recorded. Results of the feasibility analysis from the family members’ perspective are presented in Table 4.

**Table 4:** Perceived support by the family-scout at T1 as assessed by the healthy partners

Items	Mean	SD	Min	Max	*N*
I was able to rely on the family-scout when having problems with the illness of my partner.	3,36	,802	1	4	137
The family-scout supported me so that it was easier to deal with the illness of my partner.	3,11	,906	1	4	140
The family-scout was willing to listen to my problems relating to the illness of my partner.	3,57	,711	1	4	140

Note: Items were measured on a four−point Likert scale from 1 to 4 with higher scores representing higher ratings; SD: Standard Deviation; Min: Minimum; Max: Maximum

The sample of the interviewed healthy partners consisted of 9 women (56%). The mean age of the participants was 49 years. Many participants expressed a high level of satisfaction with the family-scout intervention, specifically highlighting the accessible nature of the service, the prompt support provided by family-scouts, the availability of assistance at all times [during working hours, authors’ note], and the presence of a dedicated contact person (even after the patient’s passing). They emphasized how these aspects significantly alleviated the burden on parents and facilitated task prioritization.

Interview participants underscored the holistic approach to the family system from both the family and children’s perspectives. Some reported child-centered communication with family-scouts, which focused on the well-being of the children. Support was twofold, with family-scouts engaging with the children on one hand and parents discussing their children’s well-being with the family-scouts on the other hand. This dual approach provided reassurance to parents about their children’s welfare. Participants found it beneficial that family-scouts cared for the entire family unit and continued to address the needs of each family member, even after the potential loss of the patient. Participants described receiving emotional support from family-scouts and cultivating a trusting, dependable, and close relationship with them.

While some participants expressed overall satisfaction with the support provided by family-scouts, they were unable to fully utilize the opportunities due to intermittent digital support resulting from the COVID-19 pandemic. Additionally, some families had a sufficient support system within their social network, while others did not fully grasp the potential benefits of the family-scout intervention.

Participants found home visits helpful and valued having an unbiased partner in their journey. For some, the intervention laid the groundwork for addressing family issues. However, in certain cases, specific individual problems remained unresolved. A minority of participants perceived the family-scout support as burdensome, with home visits being described as stressful and unhelpful. Some conversations were seen as overly directive, offering too much advice that couldn’t be followed or that was deemed inappropriate.

## Discussion

The aim of this work was to analyze the feasibility of the complex intervention family-scout. Special emphasis was placed on the acceptability, suitability and implementability of the intervention and the preliminary evaluation of participant responses. The feasibility of the intervention was assessed based on study monitoring and process evaluation from the family-scouts’ and the families’ perspective.

The results provide a comprehensive picture of the feasibility of the intervention. The acceptance is high and families show positive responses to the intervention: As assessed by the partners, most families feel highly supported by their family-scout; most of them feel they can rely on their family-scout and feel listened to when sharing their concerns. Especially the permanent contact person, the opportunity to get help at any time and the support even after the death of the patient is perceived as helpful. This is reflected by the professional perspective: As assessed by the family-scouts, the degree of acceptance of the intervention in the families is high; most families are relieved to accept the offer. However, the acceptability of the intervention depends on the specific functioning of the family system and the family’s motivation. The families’ burden is not solely predictive for the use of the intervention, the willingness to accept help must also be given. Some families have a pronounced functional image of themselves and do not want to abandon it, others deliberately protect their family system against help from outside their system. The family-centered arrangement of the encounters in terms of frequency, duration and mode is crucial for the acceptability. The family determines the conditions and the framework of the intervention. The family-scouts offer their continuous support; to what extent the family will make use of it depends on their decision, respectively. The acceptability and suitability also depend on the relationship between the family-scouts and the family members.

In line with the study by Inhestern et al. (2016) our results show that intervention characteristics such as a flexible structure are important facilitators of implementation. The monitoring revealed that a psycho-oncological affiliation and medical counseling for the family-scouts are needed to implement the intervention. The family-scouts themselves need a professional background in social work but benefit from psychotherapeutic supervision. These findings are in line with the origin and history of psychosocial care for cancer patients and their families as it is mainly the psychooncologists’ perspective on the unmet needs of this patient population that was the starting point for many activities in psychosocial care in oncology (Breitbart and Alici 2009). Family-scouts benefit from the psychooncological supervision in their own daily work with the families and also regarding the issue of screening for the need of an additional or subsequent psychotherapy for single family members or the whole family. In addition, the professional network is the core element for a successful intervention. The family-scout has to cooperate closely with the professional network members in order to be able to satisfy all family needs in terms of a comprehensive case- and care-management (Petermann-Meyer and Hugot 2022). The family-scout receives information both from the families themselves as well as from partners in the professional network. A unique characteristic of the intervention is that the family-scout can accompany the family in phases of inpatient treatment, during inpatient rehabilitation, follow up, and after the decease of the affected parent. Then the intervention fulfills its requirement of cross-sectoral support of all family members.

The leading decision-making body in the German health care system (Gemeinsamer Bundesausschuss) has recently approved the reimbursement of the service by the statutory health insurance based on the effectiveness results. The roll out and long-term implementation should be subject to accompanying implementation research as acceptability and feasibility are dynamic concepts and will require reconsideration as intervention are scaled to different settings (Damschroder et al. 2022). The evaluation of implementation processes and experiences in smaller hospitals or cancer centers will help to develop an implementation theory and to implement checklists that are context-specific and transferrable to different settings and organizational structures.

There are limitations to be considered in interpreting and generalizing our results. The results are based on the monitoring and survey results at two intervention sites in Germany. Participants were mainly screened and enrolled in comprehensive cancer centers. Hence, our results might not be generalizable to smaller cancer centers or hospitals. There is a selection bias towards (native) German speaking families due to the inclusion criteria. This is particularly problematic, given that the utilization of psycho-oncological services is very low in population groups with specific migration backgrounds (Singer et al. 2023). There might also be a selection bias towards families who were obviously in need for support being more likely to be screened for eligibility. The analysis of screening, initial response and the representativeness of our final study sample is subject to ongoing analyses. The family-scout teams were small and comprised of women exclusively. Additionally, the family-scouts were supervised both by an administrative study project manager and by a psycho-oncologist, respectively. These organizational structures and resources are given in the context of the family-scout effectiveness-implementation trial and might not be given in routine care in the future. Factors affecting the implementation success of health care innovations might be related to guidelines, individual health professional factors, patient factors, professional interactions, incentives and resources, capacity for organizational change, and social, political and legal factors (Flottorp et al. 2013). Our approach of assessing implementation resources did not allow the exploration of possible barriers from the perspective of possible referring healthcare professionals in oncology who were not familiar with the family-scout intervention or were not part of the effectiveness trial. The inclusion of providers who do and do not participate is recommended in feasibility studies (Forstner et al. 2019) to be able to develop a comprehensive implementation theory. These aspects will be subject to future implementation research. Additionally, the burden and restrictions caused by the COVID-19 pandemic in 2020 and 2021 have been an unexpected challenge for families (Hoppe et al. 2023), the family-scouts and the design of the intervention (Büntzel et al. 2021; Voigtländer et al. 2021). The intervention has been amended by home visits via video calls. To what extent the additional offer of video calls instead of home visits will be further used after the pandemic will become apparent in the future.

## Conclusion

The family-scout intervention is a feasible way to reduce the burden of patients with cancer having minor children and their families. First evidence for the effectiveness of the intervention has been found (Petermann-Meyer et al. 2024). In future, all families with statutory health insurance in Germany will be able to take advantage of this offer. However, different approaches should be used and combined to best meet the needs of families. These may also include support programs especially for parents (Skrabal Ross et al. 2023, 2024), or especially for children (Lysecki et al. 2024) and training programs for health care professionals in caring for families affected by parental cancer (Inhestern et al. 2019; Johannsen et al. 2020). Strengthening health care professionals’ skills to care for patients with minor children along with raising awareness of the psychosocial burden associated with a parental cancer diagnosis could improve initial support right after a cancer diagnosis, increase knowledge about and visibility of supportive structures, facilitate timely referrals of families and allow comprehensive psychosocial care for families in all settings and stages of illness.

## Additional information material

This documentary offers insights into the intervention and the experiences of affected families: https://www.youtube.com/watch?v=cLbgd86rioM.

## Data Availability

The anonymized survey and interview data of this study are available from the authors upon reasonable request.
